# Quantitation of total fatty acids in plasma and serum by GC-NCI-MS

**DOI:** 10.1016/j.clinms.2016.12.001

**Published:** 2016-12-20

**Authors:** E. Kish-Trier, E.L. Schwarz, M. Pasquali, T. Yuzyuk

**Affiliations:** a ARUP Institute for Clinical and Experimental Pathology, Salt Lake City, UT, United States; b Department of Pathology, University of Utah, Salt Lake City, UT, United States

**Keywords:** Fatty acids, Omega-3, Omega-6, Gas chromatography, Negative chemical ionization, Mass spectrometry, Pentafluorobenzyl, Triene/tetraene ratio

## Abstract

Polyunsaturated fatty acids (PUFAs), including essential omega-3 and omega-6 fatty acids, play important roles in diverse physiological and pathological processes. Diligent monitoring of PUFAs is recommended for individuals with increased risk of developing essential fatty acid deficiency (EFAD), including premature and very low birth weight infants, patients on prolonged parenteral nutrition, and those with dietary restrictions, for example due to inborn errors of metabolism. Here, we present a gas chromatography-negative chemical ionization-mass spectrometry (GC-NCI‐MS) method for the quantitation of total levels of twenty-two fatty acids (C12‐C22) in serum/plasma, including omega-3 and omage-6 PUFAs. Hydrolysis was used to release esterified fatty acids, which were analyzed by GC-NCI-MS as pentafluorobenzyl esters in selected-ion monitoring (SIM) mode. The calibration curves for all analytes had consistent slopes with R^2^ of ⩾0.990. Intra- and inter-assay precision CVs were ⩽9.0% and ⩽13.2%, respectively. Samples were found to be stable for 24 h at room temperature, at least 7 days at 4 °C, at least 75 days at −20 °C, and for three freeze/thaw cycles. No matrix effects or interferences were observed. This method offers improvements over published studies including smaller sample volume, inclusion of additional internal standards, analysis in a single injection, and use of methane reagent gas. This method could be used in a clinical laboratory setting for the diagnosis of EFAD, evaluation of nutritional status, and diet monitoring.

## Highlights

• Total C12 to C22 fatty acids in serum or plasma are quantitated by GC-NCI-MS.
• Reference ranges for total fatty acids in serum or plasma are presented.
• Method performance is comprehensively validated.

## 1 Introduction

Fatty acids (FAs), as major constituents of lipids, are involved in diverse aspects of eukaryotic cellular function such as energy storage, membrane structure and dynamics, and signal transduction [1]. The length of the alkyl chain, degree of its saturation, and position of the double bond (ω3, ω6, ω7 or ω9) confer the biological activities unique to the various FAs. Almost all polyunsaturated FAs (PUFAs) can be synthesized by the human body with the exceptions of omega-3 alpha-linolenic acid (C18:3ω3) and omega-6 linoleic acid (C18:2ω6). These can only be obtained from diet and are precursors to multiple long-chain omega-3 and omega-6 PUFAs, such as docosahexaenoic acid (DHA, C22:6ω3) and arachidonic acid (ARA, C20:4ω6), which are critical to normal growth, neurological development, vision, immune response and inflammation. The rate of conversion of alpha-linolenic and linoleic acids to long-chain, omega-3 and omega-6 PUFAs is slow and can be further limited by enzymatic insufficiency due, for example, to prematurity or severe liver damage [2], [3], [4], [5]. Deficiency in long-chain omega-3 and omega-6 PUFAs, referred to as essential fatty acid deficiency (EFAD), can result in dermatitis, poor wound healing, infection, decreased growth, impaired learning, and infertility [6], [5].

Given the clinical implications in human health, FA analysis has been an area of interest for many years [7], [8]. LC-MS/MS methods provide considerable sensitivity and selectivity [9], [10], however, the clinically validated methods for FA quantitation are predominantly GC–MS-based [11], [12]. GC provides more resolving power than LC and is well suited to profiling FAs, many of which are positional isomers. In order to decrease polarity and increase volatility, FAs are derivatized to form methyl esters (FAMEs) or pentafluorobenzyl esters (PFB-FAs) [13], [8]. Ionization of FAMEs is achieved by electron ionization (EI), whereas PFB-FAs are ionized by chemical ionization (CI). EI induces considerable fragmentation of FAMEs [13], whereas negative CI (NCI) of PFB-FAs results in little or no fragmentation, which is advantageous for quantitation [14].

Only a fraction of FAs exist in a free form, the majority are bound by proteins or esterified in higher order lipid structures, such as triacylglycerols and phospholipids [15]. Quantitation of total (free and esterified) FAs in plasma or serum is clinically useful for the diagnosis of EFAD, as well as evaluating nutritional status. Monitoring FA levels is important in individuals with increased risk of developing EFAD due to prematurity, prolonged parenteral nutrition, malabsorption or severe liver damage, as well as in patients with genetic disorders, such as cystic fibrosis and those on dietary restrictions due to inborn errors of metabolism.

Numerous methods for the quantitation of FAs in various matrices have been published, yet few include clinical validation data or reference range information. Here, we describe the development and comprehensive validation of a GC-NCI-MS method for the quantitation of total levels of 22 FAs (C12-C22) in plasma and serum. This robust and reliable method is well suited for clinical laboratory use in the diagnosis of EFAD, as well as in evaluation of nutritional status and diet monitoring.

## 2 Materials and methods

### 2.1 Reagents, chemicals, and tubes

10 N sodium hydroxide (NaOH) and concentrated hydrochloric acid (HCl) were purchased from VWR. N,N-diisopropylethylamine (DIPEA) and Pentafluorobenzyl bromide (PFB-Br) were obtained from Sigma-Aldrich. Organic solvents were HPLC grade or higher and water was Type I. Twenty-two FA standards and twelve internal standards (IS) were obtained commercially (Tables S.1 and S.2). Delipidated human serum was purchased from Golden West Biologicals (#MSG3000) or SeraCare Life Sciences (#22011). SeraCare Life Sciences serum was used in stearic acid validation experiments. All glassware used in this procedure was pre-treated with CONTRAD 70 (Decon Labs) to decrease background levels of FAs. Hydrolyses were carried out in Chomsystems tubes (#2010).

### 2.2 Calibrators and controls

Six, non-zero, calibrators were prepared in ethanol by combining twenty-two FA standards at concentrations spanning the analytical measurement range (AMR; Table S.1). A mix of twelve stable-isotope-labeled IS was prepared in ethanol. FAs, for which corresponding IS were not available, were assigned IS with similar chemistry (Table S.1). Calibration curve slope, y-intercept, and R^2^ were monitored over twelve runs to evaluate reproducibility of calibration. A Normal Control (NC), prepared from serum of normal healthy donors after ⩾12 h fasting, and a Low Normal Control (LNC), obtained by a twofold dilution with delipidated serum, were used in all validation runs to evaluate acceptability. Single use aliquots of calibrators, IS mix, NC, and LNC were stored at −80 °C until use.

### 2.3 Samples

Reference ranges were established using plasma and serum samples submitted to our laboratory and reported as normal on routine biochemical genetic tests (n = 239). In addition, specimens collected after ⩾12 h of fasting from healthy children between 6 months and 17 years of age (n = 59) and from healthy adults (n = 8) were also included in this study. A total of 306 plasma/serum samples were analyzed to establish reference ranges in three age groups: <1 month (n = 57), 1 ⩽ 12 months (n = 120), >1 year (n = 129). Reference intervals were determined using EP evaluator (Data Innovations) by non-parametric analysis and represent the central 95% (2.5–97.5%) of the population. A non-parametric Mann–Whitney test (2-tailed) was used to test differences in FA concentrations between various age groups. P-value <0.05 was considered to be significant. All samples were de-identified and used according to protocols approved by the IRB of the University of Utah. The specimens were stored at −80 °C until analysis.

### 2.4 Sample preparation

Calibrators, controls, and patient samples were extracted and derivatized according to a procedure modified from Lagerstedt et al. [11]. Briefly, 50 μl of sample was combined with 50 μl IS mix in a glass tube and hydrolyzed in the presence of 1 ml acetonitrile (ACN):6 N HCl (9:1). 1 ml MeOH:10 N NaOH (9:1) was then added and the sample was hydrolyzed again. Both hydrolyses were carried out at 100 °C for 45 min to release esterified FAs. 180 μl of 6 N HCl and 3 ml of hexane were then added to the samples, followed by vortexing for 2 min at 1200 rpm and centrifugation at room temperature (RT) for 4 min at 1200 × *g* to separate phases. The organic layer was transferred to a new glass tube and dried down under nitrogen at 37 °C. Samples were derivatized with a mix of 100 μl 10% PFB-Br and 100 μl 10% DIPEA, both in ACN, for 30 min at RT. After incubation, 20 μl 6 N HCl and 1 ml of hexane were added to each tube. Tubes were vortexed and 150 μl of the upper organic layer was transferred to an autosampler vial.

### 2.5 GC–MS conditions

Analysis was conducted with an Agilent Technologies 5977A/7890B GC–MS using helium carrier gas. 2 μl of extracted sample were delivered by split injection (220:1) onto the first of two ZB-1 ms (15 m × 0.25 mm I.D. × 0.25 μm film) columns (Phenomenex #7Eg-G001-11) connected in tandem by a purged ultimate union. Separation of FAs was achieved by temperature gradient. The oven temperature program started at 150 °C, increased to 200 °C in two minutes, followed by a ramp to 310 °C in 22 min. Columns were back-flushed post-run with 5 void volumes at 325 °C to remove non-volatile compounds. GC parameters were as follows: inlet temperature (300 °C), pulsed split mode, column 1 flow (1.2 ml/min), column 2 flow (1.24 ml/min), and MS transfer line (315 °C). FAs were ionized using NCI with methane and detected by a single quadrupole in selected-ion monitoring (SIM) mode. MS parameters were as follows: source temperature (240 °C), quadrupole temperature (150 °C), and methane flow (40%, 0.2 ml/min). Dwell times ranged from 60 to 150 ms, depending on peak width and number of analytes in the SIM group. The GC–MS system was controlled by a Agilent MassHunter Workstation.

### 2.6 Data analysis

Data were batch processed with Agilent MassHunter Quantitation. Peaks were integrated with the MQ4 algorithm, followed by visual inspection and manual adjustment, if required. Separate linear calibration curves were fitted for each analyte using 1/x weighting. The response of each analyte was normalized using IS. Analytes were reported in whole numbers. Quantitation of the 22 FAs permitted calculation of aggregate values (*i.e.*, total omega-3 and omega-6) in addition to the triene/tetraene (TT) ratio (see Section 3.7). These calculations were carried out in Microsoft Excel. Results, expressed as a percentage of total, were calculated by summing the absolute values of all FAs analyzed in this method and dividing each FA by this value (see Section 3.4).

### 2.7 Method validation

#### 2.7.1 Accuracy and precision

Accuracy of the method was determined by comparing FA concentrations in 54 patient samples between our lab and a reference lab using the method presented in Lagerstedt et al. [11]. The data were analyzed by Deming regression in EP Evaluator (Data Innovations). Intra-day precision was evaluated by extracting controls (NC, LNC) and two patient samples in triplicate on three separate days. To determine inter-day precision, control and patient samples were extracted on twelve and six different days, respectively.

#### 2.7.2 Stability studies

Control and patient samples were aliquoted and stored at −80 °C for the duration of the study. The stability of FAs at −80 °C and −20 °C was confirmed by comparing their concentrations in freshly collected and frozen samples at regular intervals throughout the study. In addition, the stability of FAs was also established at room temperature and at 4 °C. All samples from a given storage temperature were analyzed in a single batch and compared to aliquots stored at −80 °C from the initial collection. The freeze-thaw stability of FAs was established by subjecting patient samples (n = 3) to three freeze–thaw cycles. Results within ±15% of the original results met the acceptability criteria for all stability studies.

#### 2.7.3 Linearity and analytical measurement range

Analyte linearity and analytical measurement range (AMR) were determined by analyzing the recovery of FAs (measured *vs.* expected concentrations) in calibrators and in delipidated serum spiked with FA standards on twelve and four separate days, respectively. Delipidated serum was extracted in parallel to determine baseline concentrations, which were subtracted from the measured concentrations. Recovery within ±20% at the lowest concentration and ±15% at all other levels was considerate acceptable.

#### 2.7.4 Sensitivity and specificity

The analytical sensitivity of the method was evaluated by diluting the lowest calibrator (Cal1) both twofold and threefold with delipidated serum. Samples were extracted on three separate days. The limit of quantitation (LOQ) was established as the lowest concentration where the measured and expected concentrations were within 20% and CV < 20%. The lower limit of detection (LOD) was established as the concentration where peaks were observed at the correct retention time, with a signal to noise ratio >5.

#### 2.7.5 Matrix effects

To evaluate matrix contribution to IS signals, ten patient samples were extracted without IS, and the relative abundance of IS signals in these samples were compared with IS mix extracted neat. In addition, matrix effect on the recovery of FAs was investigated by spiking seven patient plasmas with FA standards at Cal3 and Cal5 concentrations (Table S.1). Analysis of analyte recovery at lower concentrations was not feasible due to high endogenous amounts of FAs. Recoveries within ±10% of the expected concentrations and CV < 15% were considered acceptable.

#### 2.7.6 Interference studies

To assess potential interferences due to hemolysis, blood was collected from three healthy donors and aliquoted. Plasma was separated immediately from one aliquot and the other aliquots were partially hemolyzed prior to plasma separation by placing tubes for up to 5 min at −80 °C. The effect of icterus on FA measurements was evaluated in delipidated serum samples with FA concentrations at Cal2, Cal4, and Cal5 levels (Table S.1) spiked with bilirubin (7.25 mg/dl final concentration) or an equal volume of control buffer (0.1 N NaOH). The use of a bilirubin concentration above the reference level is standard practice in our laboratory to rigorously evaluate test robustness. Results within ±10% of the expected concentrations indicated no significant interference effect at the concentrations studied.

#### 2.7.7 Collection conditions

Blood from ten healthy, non-fasting volunteers was collected in EDTA (lavender top), sodium heparin (green top), and serum separator (gold top) 13 × 100 mm plastic vacutainer tubes (Becton, Dickinson, and Company). Plasma and serum were separated within 45 min post-draw by centrifugation at 12,000*g* for 10 min, aliquoted and stored at −80 °C until analysis. Analyte CVs of ⩽10% between tube types was considered acceptable to establish equivalence among specimen types.

## 3 Results and discussion

### 3.1 Method improvements

Several improvements to the original method [11] were made in this study. They included reduction in sample volume and introduction of a second liquid-liquid extraction step in place of a dry down post-derivatization step, which avoided re-dissolving PFB-FAs in the final solvent, a process which is slower and may be incomplete for samples with high levels of FAs. Secondly, broad concentration ranges of FAs were acquired in a single split injection as opposed to two injections used in the original method (one splitless to detect low abundance FAs, and a second split to detect higher abundance FAs). The use of a single injection not only shortens the total run time, but also facilitates the analysis and reporting of results. These improvements, together with the use of reference standards for each of the twenty-two FAs, resulted in greater analytical performance of the assay in comparison to the original method (see Section 3.2). Lastly, sufficient sensitivity was found using methane as the NCI reagent gas instead of ammonia. Methane is preferable as it is less toxic and has fewer negative impacts on the instrument, specifically on the foreline pump; additionally, suitable ammonia gas filters for the removal of oxygen and water are unavailable.

### 3.2 Analytical performance

#### 3.2.1 GC–MS

**Fig. 1:**
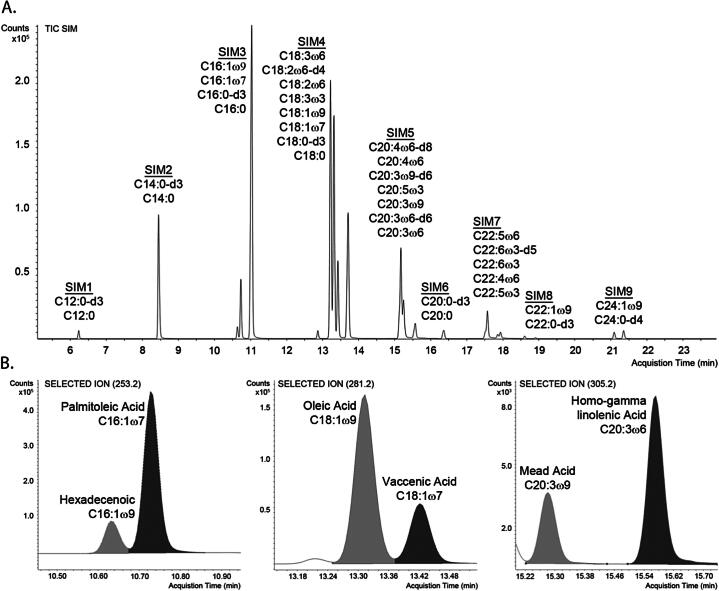
Chromatographic analysis of total fatty acids in serum/plasma by GC-NCI-MS. (A) Total ion chromatogram of 22 FAs and 12 IS mass spectral peaks detected in 9 SIM groups over a 24 min oven program. (B) Selected ion chromatograms of FA positional isomers. For selected ion chromatograms of all analytes, please see Figs. S.1 and S.2.

PFB derivatives of twenty-two FAs and twelve IS were detected in one of nine SIM groups (Fig. 1). Single peaks were observed and quantitated for all but docosenoic acid (C22:1ω9), which showed multiple peaks, presumably isomers, at the same *m*/*z* (337.3) in patient samples. Consistent with reported practices, all *m*/*z* 337.3 peaks near the docosenoic RT were integrated and included in quantitation [11]. Baseline separation was achieved for the majority of FAs (Figs. S.1 and S.2), which allowed automatic integration of the peaks using MassHunter Quantitation. Only vaccenic acid, DPAω6, and the aforementioned docosenoic peaks required manual adjustment in some patient samples.

#### 3.2.2 Accuracy and precision

Comparison of FA concentrations in patient samples using Deming regression statistics (n = 54) between our lab and an unaffiliated reference laboratory demonstrated the agreement of measurements within ±20% for the majority of FAs (17 out of 22; Table 1). Correlation coefficient (R) values for this comparison were >0.90 for all analytes, suggesting discrepancies in the measurement of these FAs are likely caused by differences in reference material used for quantitation, chromatographic separation, or integration practices. Reference levels were established for all analytes in the panel (see Section 3.5). The intra- (⩽9.0%) and inter- (⩽13.2%) assay CV% obtained with this method (Table 1) are improved compared to the original method (⩽13.2% and ⩽22.9, respectively) [11], and similar to a FAMEs method for a subset of these FAs [12]. Notably, the inter-assay precision studies were conducted under variable conditions (four operators, different reagent and GC column lots), demonstrating the method’s robustness. Calibration curve statistics from twelve separate batches run over three months showed reproducible calibration with CVs for analyte slopes ⩽10.1% and R^2^ > 0.990 for all analytes and batches.

**Table 1:** Method validation results for quantitation of total fatty acids in serum/plasma by GC-NCI-MS.

Fatty Acid	Chain	AMR/Linearity^1^ (nmol/ml)	LOQ (nmol/ml)	LOD (nmol/ml)	Precision (CV%)		Method Comparison	
					Intra-day	Inter-day^2^	Slope	R
Lauric (Dodecanoic)	C12:0	2–300	1	0.6	⩽8.4	⩽10.2 (11.1)	0.87	0.967
Myristic (Tetradecanoic)	C14:0	15–700	8	4.5	⩽3.1	⩽5.4 (4.1)	0.82	0.991
Palmitic (Hexadecanoic)	C16:0	150–6000	45	45	⩽3.3	⩽7.8 (6.2)	1.10	0.983
Stearic (Octadecanoic)	C18:0	50–2500	50	50	⩽5.6	⩽9.7 (8.8)	0.87	0.971
Arachidic (Eicosanoic)	C20:0	4–200	2	1.2	⩽3.0	⩽6.2 (6.8)	0.92	0.970
Palmitoleic (9-Hexadecenoic)	C16:1ω7	20–2000	10	6	⩽3.6	⩽7.5 (5.3)	0.83	0.985
Hexadecenoic (7-Hexadecenoic)	C16:1ω9	10–400	5	3	⩽4.8	⩽11.5 (7.3)	0.79	0.965
Vaccenic (11-Octadecenoic)	C18:1ω7	40–2000	12	12	⩽3.1	⩽11.0 (5.8)	0.77	0.946
Alpha-Linolenic (9,12,15-Octadecatrienoic)	C18:3ω3	3–600	2	0.9	⩽4.7	⩽6.7 (5.3)	0.85	0.967
EPA (5,8,11,14,17-Eicosapentaenoic)	C20:5ω3	2–2000	2	1	⩽9.0	⩽10.4 (7.9)	1.01	0.992
DPAω3 (7,10,13,16,19-Docosapentaenoic)	C22:5ω3	4–500	2	1.2	⩽4.8	⩽5.6 (5.2)	1.10	0.965
DHA (4,7,10,13,16,19-Docosahexaenoic)	C22:6ω3	10–2500	10	3	⩽3.2	⩽6.2 (5.4)	1.08	0.989
Linoleic (9,12-Octadecadienoic)	C18:2ω6	40–8000	12	12	⩽4.2	⩽5.9 (5.6)	1.09	0.963
Gamma-Linolenic (6,9,12-Octadecatrienoic)	C18:3ω6	2–400	2	0.6	⩽4.0	⩽7.8 (6.1)	0.79	0.946
Homo-Gamma-Linolenic (8,11,14-Eicosatrienoic)	C20:3ω6	2–500	1	0.6	⩽3.0	⩽6.4 (4.4)	1.48	0.954
Arachidonic (5,8,11,14-Eicosatetraenoic)	C20:4ω6	70–3500	70	21	⩽2.7	⩽6.3 (5.5)	1.19	0.961
DTA (7,10,13,16-Docosatetraenoic)	C22:4ω6	1–200	1	1	⩽4.0	⩽7.1 (4.5)	1.01	0.955
DPAω6 (4,7,10,13,16-Docosapentaenoic)	C22:5ω6	1–200	1	1	⩽6.1	⩽5.8 (5.2)	1.01	0.974
Oleic (9-Octadecenoic)	C18:1ω9	150–5000	75	45	⩽3.6	⩽7.0 (4.3)	1.15	0.970
Mead (5,8,11-Eicosatrienoic)	C20:3ω9	1–200	0.5	0.5	⩽8.9	⩽13.2 (12.2)	1.20	0.965
Docosenoic (13-Docosenoic)	C22:1ω9	2–100	1	0.6	⩽5.8	⩽12.9 (3.6)	0.70	0.907
Nervonic (15-Tetracosenoic)	C24:1ω9	8–250	2.4	2.4	⩽5.2	⩽5.3 (7.1)	0.99	0.934

1 Analytical measurement range (AMR).

2 Inter-day precision for QC samples and patient samples (parentheses).

#### 3.2.3 Linearity, LOQ and LOD

The method was linear throughout the calibration range, and LOQs for each analyte are ⩽Cal1 (Table 1 and S.1). Established AMRs have sufficient sensitivity and linearity to accommodate reference ranges for all FAs. Moreover, FAs in concentrations above the upper limit of linearity can be reliably measured in patient samples by injecting extracts after threefold dilution with hexane (data not shown), thus expanding the reportable range of FAs important for clinical management of patients on dietary therapies.

### 3.3 Matrix effects and interference

FAs in patient samples are quantitated using ethanol-based calibrators due to the insolubility of free FAs in serum/plasma matrix. Therefore, potential matrix effects were evaluated by spiking seven plasma samples with FA standards at Cal3 and Cal5 concentrations (Table S.3). The mean for the recovery of FAs was within ±9% of the expected concentrations with precision ⩽5.7%, suggesting a negligible matrix effect on quantitation of FAs. Influence of matrix constituents on IS signal is ⩽5%, indicating the method also has sufficient matrix selectivity. Icteric samples (n = 3) were acceptable for testing: FA concentrations in samples spiked with bilirubin were within ±10% of their expected values. In contrast, severe hemolysis (>1.5 g/L of hemoglobin) resulted in decreased levels of several C18 PUFAs (Fig. S.3A) and increased concentrations of C22 PUFAs (Fig. S.3B), which were likely released from blood cell membranes during the freeze/thaw cycle. Therefore, severely hemolyzed samples (>1.5 g/L of hemoglobin) are not acceptable for testing.

### 3.4 Specimen collection and analyte stability

Results from sample collection tube comparisons indicate that plasma (EDTA or Heparin) and serum are all acceptable for the quantitation of FAs. The average CVs between results from ten patients, each drawn in EDTA, heparin and serum collection tubes, were consistently ⩽8.1%. Analyte stability in patient samples at commonly encountered storage temperatures was found to be 24 h at RT, at least 7 days at 4 °C, and at least 75 days at −20 °C (Table S.4), which is in agreement with previous studies [11], [12]. Analytes were found to be stable for at least three freeze-thaw cycles, consistent with published observations (Table S.4) [11].

### 3.5 Fatty acid reporting

By convention, FA values are generally reported as percentages of the total FA concentration [6]. The method described here reports FA concentrations as absolute values (nmol/ml). To compare the two reporting schemes, the correlation coefficients between FA values expressed in absolute (nmol/ml) and relative (% total) terms for 415 plasma and serum samples were calculated (Table S.5). Reference samples (see Section 3.5), as well as clinical samples, with FA concentrations outside the normal ranges were included in the analysis. Correlation (R) ranged from 0.91 (EPA) to 0.21 (stearic acid), with generally lower correlations for higher abundance FAs (Table S.5, Fig. S.4). Importantly, 6 of 10 omega-3 and omega-6 fatty acids, including linoleic acid and arachidonic acid, exhibited only modest correlation between absolute and relative concentrations (r < 0.80; Fig. S.4), which could lead to inconsistencies in clinical interpretation. Several other studies have noted the discrepancy between absolute and relative values [11], [16], [17], with some finding statistically significant differences between control and test groups only when using absolute concentration [16]. A more recent study also reported that the correlation of absolute versus relative values for a given FA is inversely related to abundance in red blood cells [18].

### 3.6 Reference ranges

FA reference ranges were established in three gender-nonspecific age groups: <1 month (n = 57), 1 month ⩽ 12 months (n = 120), >1 year (n = 129) (Table 2). The differences in FA concentrations between different age groups were statistically significant, p < 0.05 (see Section 2.7.6). For the majority of analytes, ranges are in agreement with a previous study [11], although some diverge, possibly reflecting differences in FA quantitation, as well as the selection of samples used for the establishment of reference ranges. For example, the higher range of DHA concentrations reported here may reflect the widespread supplementation of food products, including infant formulas with DHA, as compared to fifteen years ago [19]. In both the previous and current studies, the fasting status for a majority of reference samples was unknown, which may have contributed to bias in the upper-normal range. Yet, the reference ranges for the >1 year group found here are similar to recent studies where all participants were fasting [17], [20]. Reference ranges for several cumulative values are presented in Table 2. Totals of omega-3, omega-6, PUFAs, monounsaturated, saturated, and all fatty acids are calculated from absolute values to aid in evaluation of overall nutritional status.

**Table 2:** Reference ranges for total fatty acids in plasma determined by the method.

Fatty acid (nmol/ml)	<1 month	1 month ⩽ 12 months	>1 year
Lauric (Dodecanoic)	4–360	4–360	1–200
Myristic (Tetradecanoic)	20–420	20–420	20–520
Palmitic (Hexadecanoic)	1090–3840	1090–3840	1090–3840
Stearic (Octadecanoic)	280–1250	280–1250	280–1250
Arachidic (Eicosanoic)	11–46	11–46	8–43
Palmitoleic (9-Hexadecenoic)	50–590	20–470	35–580
Hexadecenoic (7-Hexadecenoic)	14–95	14–95	14–95
Vaccenic (11-Octadecenoic)	65–280	65–280	50–250
Alpha-Linolenic (9,12,15-Octadecatrienoic)	5–150	20–200	20–200
EPA (5,8,11,14,17-Eicosapentaenoic)	5–90	5–90	8–130
DPAω3 (7,10,13,16,19-Docosapentaenoic)	7–50	7–50	13–75
DHA (4,7,10,13,16,19-Docosahexaenoic)	75–350	75–350	45–365
Linoleic (9,12-Octadecadienoic)	380–3000	1240–3890	1210–4300
Gamma-Linolenic (6,9,12-Octadecatrienoic)	7–50	7–50	10–120
Homo-Gamma-Linolenic (8,11,14-Eicosatrienoic)	30–240	30–240	45–340
Arachidonic (5,8,11,14-Eicosatetraenoic)	340–1090	340–1090	310–1420
DTA (7,10,13,16-Docosatetraenoic)	10–40	10–40	10–40
DPAω6 (4,7,10,13,16-Docosapentaenoic)	6–65	3–30	6–55
Oleic (9-Octadecenoic)	740–3900	740–3900	740–3900
Mead (5,8,11-Eicosatrienoic)	3–50	1–32	1–35
Docosenoic (13-Docosenoic)	1–10	1–10	1–10
Nervonic (15-Tetracosenoic)	30–160	30–160	35–145

Calculated Values (μmol/ml)			
Total ω6	1.0–4.4	1.5–5.1	1.8–5.7
Total ω3	0.12–0.50	0.14–0.53	0.12–0.55
Total Polyunsaturated	1.1–4.8	1.7–5.5	2.1–6.2
Total Saturated	1.5–5.3	1.5–5.3	1.5–5.3
Total Monounsaturated	0.9–4.7	0.9–4.7	0.9–4.7
Total FA (sum of all FAs in panel)	4.5–15.0	4.5–15.0	4.5–15.0
Triene/tetraene Ratio	0.006–0.052	0.002–0.046	0.004–0.051

### 3.7 Triene/tetraene ratio

The ratio of mead acid (C20:3ω9) to arachidonic acid (C20:4ω6), known as the triene/tetraene (TT) ratio (Holman index), is a useful indicator of EFAD status [21]. Due to reduced levels of omega-3 and omega-6 FAs in patients with EFAD, augmented metabolism of non-essential oleic acid (C18:1ω9) leads to increased levels of omega-9 mead acid and, consequently, to an elevated TT ratio. Here, we find reference TT ratios of 0.002 to 0.052, which are consistent with other reports [11], [22], but far from a previously cited value of normal being up to 0.4 [21], [23], [24], [25]. This discrepancy has been suggested to arise from the inadequate separation of mead acid [24] and may also be related to the availability and quality of reference material at the time of a given study.

**Fig. 2:**
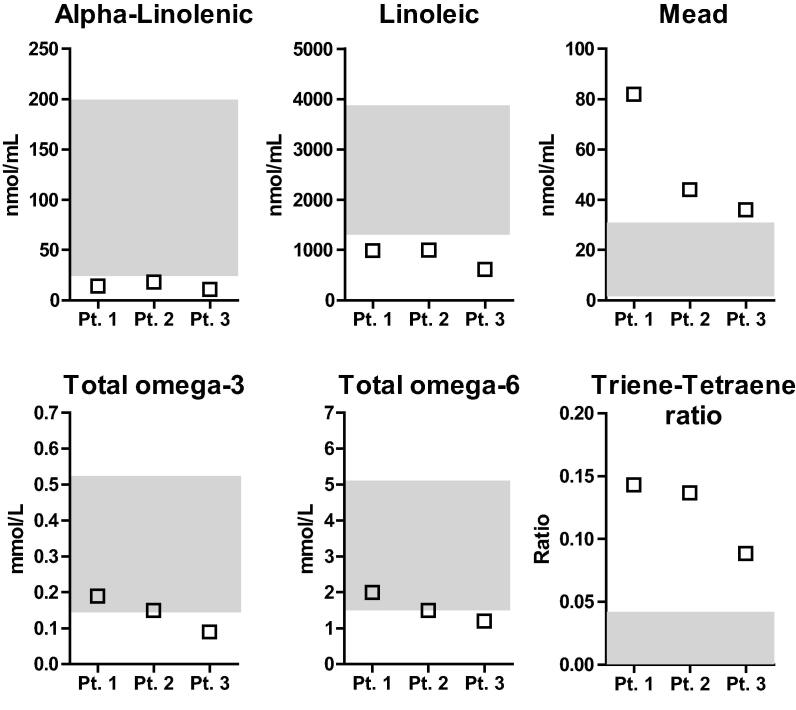
Evidence of EFAD in patient samples. Three patients showed the pattern consistent with EFAD deficiency: elevated concentrations of mead acid, increased triene/tetraene ratios and reduced levels of omega-3 alpha-linolenic and omega-6 linoleic acids. Shaded region comprises normal range.

Notably, TT ratio is not enough to discern EFAD in some patients on parenteral nutrition where lipid emulsions, high in oleic acid, may artificially elevate the TT ratio [25]. Therefore, the levels of individual omega-3 and omega-6 FAs should also be taken into consideration for an EFAD diagnosis as illustrated in Fig. 2, which shows a pattern consistent with EFAD deficiency in three patients evaluated in the method comparison (Section 3.2.2): low concentrations of alpha-linolenic and linoleic acids in addition to elevated TT ratio.

## 4 Conclusion

In summary, we have developed and validated a GC-NCI-MS method for quantitation of total fatty acids (C12-C22) in serum and plasma, including the essential omega-3 and omega-6 PUFAs. The method is robust, with single injection analysis, improved precision, and broad analytical measurement ranges. Furthermore, fatty acids are quantitated as absolute values using this method, which eliminates the influence of changes in overall fatty acid composition. An efficient and accurate method of fatty acid analysis is clinically useful for evaluating nutritional status and detecting EFAD.

## Conflict of interest

The authors wish to confirm that there are no known conflicts of interest associated with this publication and there has been no significant financial support for this work that could have influenced its outcome.
