# Supplementary material for: Quantitation of total fatty acids in plasma and serum by GC-NCI-MS
Source: Clin Mass Spectrom. 2016 Dec 20;2:11–7. doi: 10.1016/j.clinms.2016.12.001 (PMC11322783; doi:10.1016/j.clinms.2016.12.001)
Supplement: Supplementary data 1 [file mmc1.docx]

Figure S.1 – Selected ion chromatograms of analytes and internal standards for calibrator





Selected ion chromatograms of fatty acids and internal standards, presented in order of retention time, from analysis of calibrator level 4.
