# Supplementary material for: Quantitation of total fatty acids in plasma and serum by GC-NCI-MS
Source: Clin Mass Spectrom. 2016 Dec 20;2:11–7. doi: 10.1016/j.clinms.2016.12.001 (PMC11322783; doi:10.1016/j.clinms.2016.12.001)
Supplement: Supplementary data 2 [file mmc2.docx]

Figure S.2 – Selected ion chromatograms of analytes and internal standards for patient sample





Selected ion chromatograms of fatty acids and internal standards, presented in order of retention time, from analysis of typical patient plasma sample. Docosenoic acid is quantitated as the sum of all *m/z* 337.3 peaks near the 13-docosenoic acid standard peak, consistent with reported practices [11].
