# Supplementary material for: Quantitation of total fatty acids in plasma and serum by GC-NCI-MS
Source: Clin Mass Spectrom. 2016 Dec 20;2:11–7. doi: 10.1016/j.clinms.2016.12.001 (PMC11322783; doi:10.1016/j.clinms.2016.12.001)
Supplement: Supplementary data 3 [file mmc3.docx]

Figure S.3 – Effect of sample hemolysis on fatty acid analysis by this method

Effect of hemolysis on the concentrations of A) C16-C20 PUFA, shown as group averages, error bars represent standard deviations, and B) C22-24 omega-3, 6, 9 PUFAs. Changes are shown as % of control values.
