# Supplementary material for: Quantitation of total fatty acids in plasma and serum by GC-NCI-MS
Source: Clin Mass Spectrom. 2016 Dec 20;2:11–7. doi: 10.1016/j.clinms.2016.12.001 (PMC11322783; doi:10.1016/j.clinms.2016.12.001)
Supplement: Supplementary data 4 [file mmc4.docx]

Figure S.4 - Correlation between absolute (nmol/ml) and relative (% total) values for omega-3 and omega-6 FAs
