# Supplementary material for: Quantitation of total fatty acids in plasma and serum by GC-NCI-MS
Source: Clin Mass Spectrom. 2016 Dec 20;2:11–7. doi: 10.1016/j.clinms.2016.12.001 (PMC11322783; doi:10.1016/j.clinms.2016.12.001)
Supplement: Supplementary data 5 [file mmc5.docx]

Table S.1 – Fatty acids, internal standards, and calibration levels

| Fatty Acid | MW | IS | Cal6 | Cal5 | Cal4 | Cal3 | Cal2 | Cal1 | IS |
| --- | --- | --- | --- | --- | --- | --- | --- | --- | --- |
|  |  |  | (nmol/ml) | | | | | | |
| Lauric (Dodecanoic) | 200.3 | Lauric-d3 | 300 | 151 | 50 | 20 | 5 | 2 | 20 |
| Myristic (Tetradecanoic) | 228.4 | Myristic-d3 | 700 | 358 | 125 | 56 | 22 | 15 | 600 |
| Palmitic (Hexadecanoic) | 256.4 | Palmitic-d3 | 6000 | 3075 | 1086 | 501 | 209 | 150 | 520 |
| Stearic (Octadecanoic) | 284.5 | Stearic-d3 | 2500 | 1275 | 442 | 197 | 75 | 50 | 200 |
| Arachidic (Eicosanoic) | 312.5 | Arachidic-d3 | 200 | 102 | 35 | 16 | 6 | 4 | 20 |
| Palmitoleic (9-Hexadecenoic) | 254.4 | Palmitic-d3 | 2000 | 1010 | 337 | 139 | 40 | 20 | 520 |
| Hexadecenoic (7-Hexadecenoic) | 254.4 | Palmitic-d3 | 400 | 205 | 72 | 33 | 14 | 10 | 520 |
| Vaccenic (11-Octadecenoic) | 282.5 | Stearic-d3 | 2000 | 1020 | 354 | 158 | 60 | 40 | 200 |
| Alpha-Linolenic (9,12,15-Octadecatrienoic) | 278.4 | Linoleic-d4 | 600 | 302 | 99 | 39 | 9 | 3 | 150 |
| EPA (5,8,11,14,17-Eicosapentaenoic) | 302.5 | Arachidonic-d8 | 2000 | 1001 | 322 | 122 | 22 | 2 | 210 |
| DPAω3 (7,10,13,16,19-Docosapentaenoic ) | 330.5 | DHA-d5 | 500 | 252 | 83 | 34 | 9 | 4 | 100 |
| DHA (4,7,10,13,16,19-Docosahexaenoic ) | 328.5 | DHA-d5 | 2500 | 1255 | 408 | 159 | 35 | 10 | 100 |
| Linoleic (9,12-Octadecadienoic) | 280.4 | Linoleic-d4 | 8000 | 4020 | 1314 | 518 | 120 | 40 | 150 |
| Gamma-Linolenic (6,9,12-Octadecatrienoic) | 278.4 | Linoleic-d4 | 400 | 201 | 66 | 26 | 6 | 2 | 150 |
| Homo-Gamma-Linolenic (8,11,14-Eicosatrienoic) | 306.5 | Homo-Gamma-Linolenic-d6 | 500 | 251 | 82 | 32 | 7 | 2 | 60 |
| Arachidonic (5,8,11,14-Eicosatetraenoic) | 304.5 | Arachidonic-d8 | 3500 | 1785 | 619 | 276 | 104 | 70 | 210 |
| DTA (7,10,13,16-Docosatetraenoic) | 332.5 | DHA-d5 | 200 | 101 | 33 | 13 | 3 | 1 | 100 |
| DPAω6 (4,7,10,13,16-Docosapentaenoic ) | 330.5 | DHA-d5 | 200 | 101 | 33 | 13 | 3 | 1 | 100 |
| Oleic (9-Octadecenoic) | 282.5 | Stearic-d3 | 5000 | 2575 | 926 | 441 | 199 | 150 | 200 |
| Mead (5,8,11-Eicosatrienoic) | 306.5 | Mead-d6 | 200 | 101 | 33 | 13 | 3 | 1 | 60 |
| Docosenoic (13-Docosenoic) | 338.6 | Behenic-d3 (C22:0) | 100 | 51 | 18 | 8 | 3 | 2 | 6 |
| Nervonic (15-Tetracosenoic) | 366.6 | Lignoceric-d4 (C24:0) | 250 | 129 | 47 | 23 | 10 | 8 | 50 |

Table S.2 – Commercial sources of fatty acids and internal standards

| Fatty Acid | Supplier | Product No. |
| --- | --- | --- |
| Lauric (Dodecanoic) | Nu-Chek Prep, Inc. | N-12-A |
| Myristic (Tetradecanoic) | Nu-Chek Prep, Inc. | N-14-A |
| Palmitic (Hexadecanoic) | Nu-Chek Prep, Inc. | N-16-A |
| Stearic (Octadecanoic) | Nu-Chek Prep, Inc. | N-18-A |
| Arachidic (Eicosanoic) | Nu-Chek Prep, Inc. | N-20-A |
| Palmitoleic (9-Hexadecenoic) | Nu-Chek Prep, Inc. | U-40-A |
| Hexadecenoic (7-Hexadecenoic) | Cayman Chemical | 10007290 |
| Vaccenic (11-Octadecenoic) | Nu-Chek Prep, Inc. | U-48-A |
| Alpha-Linolenic (9,12,15-Octadecatrienoic) | Nu-Chek Prep, Inc. | U-62-A |
| EPA (5,8,11,14,17-Eicosapentaenoic) | Nu-Chek Prep, Inc. | U-99-A |
| DPAω3 (7,10,13,16,19-Docosapentaenoic ) | Nu-Chek Prep, Inc. | U-101-A |
| DHA (4,7,10,13,16,19-Docosahexaenoic ) | Nu-Chek Prep, Inc. | U-84-A |
| Linoleic (9,12-Octadecadienoic) | Nu-Chek Prep, Inc. | U-59-A |
| Gamma-Linolenic (6,9,12-Octadecatrienoic) | Nu-Chek Prep, Inc. | U-63-A |
| Homo-Gamma-Linolenic (8,11,14-Eicosatrienoic) | Nu-Chek Prep, Inc. | U-69-A |
| Arachidonic (5,8,11,14-Eicosatetraenoic) | Nu-Chek Prep, Inc. | U-71-A |
| DTA (7,10,13,16-Docosatetraenoic) | Nu-Chek Prep, Inc. | U-83-A |
| DPAω6 (4,7,10,13,16-Docosapentaenoic ) | Nu-Chek Prep, Inc. | U-102-A |
| Oleic (9-Octadecenoic) | Nu-Chek Prep, Inc. | U-46-A |
| Mead (5,8,11-Eicosatrienoic) | Cayman Chemical | 90190 |
| Docosenoic (13-Docosenoic) | Nu-Chek Prep, Inc. | U-79-A |
| Nervonic (15-Tetracosenoic) | Nu-Chek Prep, Inc. | U-88-A |
| Internal Standard | Supplier | Product No. |
| Lauric (Dodecanoic)-d3 | CDN Isotopes | D-4027 |
| Myristic (Tetradecanoic)-d3 | CDN Isotopes | D-3604 |
| Palmitic (Hexadecanoic)-d3 | CDN Isotopes | D-1655 |
| Stearic (Octadecanoic)-d3 | CDN Isotopes | D-1825 |
| Arachidic (Eicosanoic)-d3 | CDN Isotopes | D-5254 |
| Linoleic (9,12-Octadecadienoic)-d4 | Cayman Chemical | 390150 |
| DHA (4,7,10,13,16,19-Docosahexaenoic )-d5 | Cayman Chemical | 10005057 |
| Homo-Gamma-Linolenic (8,11,14-Eicosatrienoic)-d6 | Cayman Chemical | 10458 |
| Arachidonic (5,8,11,14-Eicosatetraenoic)-d8 | Cayman Chemical | 390010 |
| Mead (5,8,11-Eicosatrienoic)-d6 | Cayman Chemical | 10742 |
| Behenic (Docosanoic) Acid-d3 | CDN Isotopes | D-5708 |
| Lignoceric (Tetracosanoic) Acid-d4 | CDN Isotopes | D-6167 |

Tables S.3 – Recovery of fatty acids spiked into seven different patient samples at two concentrations

| Fatty acid | Mean % (n=14) | SD | CV% |
| --- | --- | --- | --- |
|  |  |  |  |
| Lauric (Dodecanoic) | 97 | 2.8 | 2.9 |
| Myristic (Tetradecanoic) | 100 | 3.1 | 3.1 |
| Palmitic (Hexadecanoic) | 97 | 3.3 | 3.4 |
| Stearic (Octadecanoic) | 97 | 2.7 | 2.8 |
| Arachidic (Eicosanoic) | 101 | 3.4 | 3.4 |
| Palmitoleic (9-Hexadecenoic) | 92 | 4.0 | 4.3 |
| Hexadecenoic (7-Hexadecenoic) | 98 | 2.5 | 2.5 |
| Vaccenic (11-Octadecenoic) | 94 | 3.4 | 3.6 |
| Alpha-Linolenic (9,12,15-Octadecatrienoic) | 104 | 4.4 | 4.2 |
| EPA (5,8,11,14,17-Eicosapentaenoic) | 101 | 5.7 | 5.7 |
| DPAω3 (7,10,13,16,19-Docosapentaenoic) | 91 | 3.0 | 3.3 |
| DHA (4,7,10,13,16,19-Docosahexaenoic) | 101 | 2.7 | 2.7 |
| Linoleic (9,12-Octadecadienoic) | 100 | 4.1 | 4.1 |
| Gamma-Linolenic (6,9,12-Octadecatrienoic) | 100 | 3.6 | 3.6 |
| Homo-Gamma-Linolenic (8,11,14-Eicosatrienoic) | 100 | 2.1 | 2.1 |
| Arachidonic (5,8,11,14-Eicosatetraenoic) | 99 | 1.2 | 1.2 |
| DTA (7,10,13,16-Docosatetraenoic) | 100 | 1.7 | 1.7 |
| DPAω6 (4,7,10,13,16-Docosapentaenoic) | 99 | 3.1 | 3.1 |
| Oleic (9-Octadecenoic) | 98 | 3.0 | 3.0 |
| Mead (5,8,11-Eicosatrienoic) | 100 | 2.6 | 2.6 |
| Docosenoic (13-Docosenoic) | 95 | 4.5 | 4.8 |
| Nervonic (15-Tetracosenoic) | 103 | 3.7 | 3.6 |

Tables S.4 – Fatty acid stability at common storage temperatures and after three freeze-thaw cycles*

| Fatty acid | RT Day0/24hrs (Ave. %; n=3) | 4 ^o^C Day0/Day7 (Ave. %; n=3) | -20 ^o^C Day0/Day75 (Ave. %; n=3) | FT-0/FT-3 (Ave. %; n=3) |
| --- | --- | --- | --- | --- |
| Lauric (Dodecanoic) | 95 | 100 | 94 | 115 |
| Myristic (Tetradecanoic) | 96 | 100 | 94 | 101 |
| Palmitic (Hexadecanoic) | 98 | 100 | 95 | 105 |
| Stearic (Octadecanoic) | 96 | 102 | 94 | 102 |
| Arachidic (Eicosanoic) | 104 | 101 | 94 | 100 |
| Palmitoleic (9-Hexadecenoic) | 94 | 101 | 93 | 103 |
| Hexadecenoic (7-Hexadecenoic) | 95 | 99 | 94 | 101 |
| Vaccenic (11-Octadecenoic) | 95 | 100 | 96 | 99 |
| Alpha-Linolenic (9,12,15-Octadecatrienoic) | 98 | 98 | 93 | 100 |
| EPA (5,8,11,14,17-Eicosapentaenoic) | 94 | 98 | 93 | 102 |
| DPAω3 (7,10,13,16,19-Docosapentaenoic) | 97 | 103 | 91 | 103 |
| DHA (4,7,10,13,16,19-Docosahexaenoic) | 97 | 101 | 92 | 99 |
| Linoleic (9,12-Octadecadienoic) | 97 | 100 | 94 | 102 |
| Gamma-Linolenic (6,9,12-Octadecatrienoic) | 95 | 99 | 93 | 97 |
| Homo-Gamma-Linolenic (8,11,14-Eicosatrienoic) | 95 | 100 | 93 | 101 |
| Arachidonic (5,8,11,14-Eicosatetraenoic) | 97 | 99 | 93 | 102 |
| DTA (7,10,13,16-Docosatetraenoic) | 92 | 106 | 91 | 106 |
| DPAω6 (4,7,10,13,16-Docosapentaenoic) | 92 | 102 | 93 | 102 |
| Oleic (9-Octadecenoic) | 100 | 100 | 94 | 102 |
| Mead (5,8,11-Eicosatrienoic) | 93 | 96 | 91 | 103 |
| Docosenoic (13-Docosenoic) | 100 | 100 | 97 | 101 |
| Nervonic (15-Tetracosenoic) | 93 | 102 | 98 | 107 |

***shown as % of control values (at -80 ^0^C); RT-room temperature

Table S.5 – Absolute vs. relative reporting (n = 415)

| Fatty acid | R |
| --- | --- |
| Lauric (Dodecanoic) | 0.90 |
| Myristic (Tetradecanoic) | 0.84 |
| Palmitic (Hexadecanoic) | 0.35 |
| Stearic (Octadecanoic) | 0.21 |
| Arachidic (Eicosanoic) | 0.34 |
| Palmitoleic (9-Hexadecenoic) | 0.81 |
| Hexadecenoic (7-Hexadecenoic) | 0.68 |
| Vaccenic (11-Octadecenoic) | 0.58 |
| **Alpha-Linolenic (9,12,15-Octadecatrienoic)** | **0.81** |
| **EPA (5,8,11,14,17-Eicosapentaenoic)** | **0.91** |
| **DPAω3 (7,10,13,16,19-Docosapentaenoic)** | **0.73** |
| **DHA (4,7,10,13,16,19-Docosahexaenoic)** | **0.74** |
| **Linoleic (9,12-Octadecadienoic)** | **0.46** |
| **Gamma-Linolenic (6,9,12-Octadecatrienoic)** | **0.83** |
| **Homo-Gamma-Linolenic (8,11,14-Eicosatrienoic)** | **0.70** |
| **Arachidonic (5,8,11,14-Eicosatetraenoic)** | **0.49** |
| **DTA (7,10,13,16-Docosatetraenoic)** | **0.54** |
| **DPAω6 (4,7,10,13,16-Docosapentaenoic)** | **0.81** |
| Oleic (9-Octadecenoic) | 0.68 |
| Mead (5,8,11-Eicosatrienoic) | 0.90 |
| Docosenoic (13-Docosenoic) | 0.79 |
| Nervonic (15-Tetracosenoic) | 0.57 |

*Omega-3 and omega-6 fatty acids are in bold*
